# Asthma Is Associated with Back Pain and Migraine—Results of Population-Based Case–Control Study

**DOI:** 10.3390/jcm12227107

**Published:** 2023-11-15

**Authors:** Natalia Gutiérrez-Albaladejo, Ana López-de-Andrés, Natividad Cuadrado-Corrales, Romana Albaladejo-Vicente, Rosa Villanueva-Orbaiz, Francisco Carricondo, Barbara Romero-Gomez, Rodrigo Jiménez-García, Napoleon Perez-Farinos

**Affiliations:** 1 Allergy Department, Hospital Universitario Fundación de Alcorcón, Alcorcón, 28922 Madrid, Spain; natagu01@ucm.es; 2 Department of Public Health and Maternal & Child Health, Faculty of Medicine, Universidad Complutense de Madrid, 28040 Madrid, Spain; mariancu@ucm.es (N.C.-C.); ralbadal@ucm.es (R.A.-V.); mrvillan@ucm.es (R.V.-O.); rodrijim@ucm.es (R.J.-G.); 3 Department of Immunology, Laboratory of Neurobiology of Hearing (UCM 910915), Ophthalmology and Otorhinolaryngology, Faculty of Medicine, University Complutense, IdISSC, 28040 Madrid, Spain; fjcarric@ucm.es (F.C.); brgomez@ucm.es (B.R.-G.); 4 EpiPHAAN Research Group, School of Health Sciences, University of Málaga—Instituto de Investigación Biomédica en Málaga (IBIMA), 29010 Málaga, Spain; napoleon.perez@uma.es

**Keywords:** asthma, chronic neck pain, chronic low back pain, migraine, surveys, epidemiology

## Abstract

(1) Background: Worldwide, asthma, back pain, and migraine are major public health problems due to their high prevalence, effect on the quality of life, and huge economic costs. The association of asthma with an increased risk of these types of pain has been suggested; however, no conclusive results have been obtained to date. The aims of our study were (1) to describe and compare the prevalence of three types of pain localization, namely migraine or frequent headaches (MFH), chronic neck pain (CNP), and chronic low back pain (CLBP), in adults with and without asthma in Spain during the years 2014 and 2020 and (2) to identify which variables were associated with the presence of these types of pain in adults with asthma. (2) Methods: A cross-sectional study and a case–control study were conducted. The 2014 and 2020 European Health Interview Surveys for Spain were used as the data source. (3) Results: A total of 2463 individuals were interviewed and had self-reported asthma. In this group, the prevalence of pain was high, with CLBP (30.9%) being the most common, followed by CNP (26.7%) and MFH (13.3%). All types of pain remained stable from 2014 to 2020. In both surveys, the women with asthma reported a remarkably higher prevalence of all the types of pain analyzed than the men with asthma. After matching by age and sex, the prevalence of all pain types was significantly higher in the patients with asthma than in the matched individuals without asthma. Multivariable adjustment showed that asthma increased the likelihood of CNP by 1.45 times (OR 1.45; 95% CI 1.19–1.76), that of CLBP by 1.37 times (OR 1.37; 95% CI 1.11–1.64), and that of MFH by 1.19 times (OR 1.19; 95% CI 1.02–1.51). The three types of pain analyzed were associated with the female sex and worse self-rated health. (4) Conclusions: Among the men and women with asthma, the prevalence of all the pain types was high and remained stable over time. The prevalence was higher and the severity was greater among the women with asthma than among the men with asthma. The prevalence of any pain was significantly higher in people with asthma than in the sex–age-matched individuals without asthma. Multivariable analysis showed that the variables associated with the reporting of the three types of pain in people with asthma were female sex, worse self-reported health, and self-reported mental illness.

## 1. Introduction

Asthma is a very prevalent chronic disease, affecting 262 million people and causing 461,000 deaths in 2019 [1]. Among Spanish adults, it is estimated that 3.9% suffer from asthma [2].

Worldwide, asthma, back pain, and migraine are considered important public health problems due to their high prevalence, effect on the quality of life, and huge economic costs [1,3,4,5,6,7,8,9]. The association of asthma with an increased risk of these types of pain has been suggested; however, no conclusive results have been obtained to date [3,4,5,6,7].

Migraine is a neurovascular disorder that affects over 1 billion people and is the second highest cause of disability worldwide. It is more common in women than in men [8,9]. Asthma has been considered a “pulmonary migraine” [10,11], as both conditions share a similar pathophysiology, with inflammation and activation of the airways or blood vessels mediated by the smooth muscle [4]. A recent study found that the risk of chronic migraine increased with the number of asthma symptoms [5].

The lifetime prevalence of low back pain (LBP) is estimated to reach 84%, ranking first among the 291 conditions with the highest number of years spent living with disability [1,3]. It is more prevalent in women and in people aged 40–80 years [4]. People with LBP are more susceptible to diaphragm fatigue than healthy people [3,4].

Asthma patients release higher amounts of pro-inflammatory cytokines (TNF-a, IL-8, and IL-1), which play an important role in LBP [3]. Both LBP and headache have been associated with inflammatory processes, as the systemic inflammation seen in asthma can trigger inflammatory responses in the vascular system of the brain and contribute to the onset of headache [3,4,5]. In addition, some medications used in the treatment of asthma, such as inhaled beta-agonists, may have adverse effects, including headache [5,6].

Neck pain (NP) is one of the most common musculoskeletal disorders, with an estimated prevalence of around 10–20% in adults [7]. Few studies have evaluated the association between asthma and NP [10,11,12,13,14]. A recent study suggests that patients with asthma have a higher risk of NP over time [12], and it offers several explanations for this hypothesis. First, the frequent and intense coughing, which sometimes occurs during asthma attacks, can cause muscle tension in the neck area, leading to pain. Second, the hypertension of the accessory respiratory muscles that connect the neck and thoracic cage can increase the risk of NP [11,12,13]. Finally, chronic neck and shoulder pain is significantly more severe in patients with asthma than in those who do not have asthma [14].

The prevalence of NP, LBP, and migraine has been reported to be consistently higher in women than in men among those with and without asthma [2,3,4,5,6,7]. The reason for this finding remains unknown, although increased inflammation due to the estrogen response and rapid post-menopausal degeneration of the spine have been suggested [5].

The association between asthma and these pain conditions is significant from a clinical and public health perspective. From a clinical perspective, the association is relevant as pain management among patients with asthma must be carefully controlled. For example, analgesics can produce important adverse events, and beta-blockers, which are effective for migraine attack prophylaxis, are not recommended [15,16,17]. Regarding the public health perspective, concomitant pain and asthma can result in an important loss in the quality of life and an increase in the use of health care services and in social costs [4,5,11,15,16,17].

In Spain, the estimated cost of asthma was EUR 1480 million a year in 2009. More recent studies have calculated a mean total annual unit cost of EUR 5493. The greatest components were days off work and loss of productivity (31.8%), hospitalizations (18.2%), and medical treatments (16.2%) [18,19]. No data are available on the contribution of chronic pains to these costs among people with asthma. However, in Spain it is estimated that chronic pains cost around EUR 16 billion a year, mainly due to LBP and NP. Therefore, the association of asthma and chronic pain is surely causing a relevant burden in our country [20]. Public health policies must be implemented to reduce the health and economic burden of these conditions.

It is therefore important to investigate the association between asthma and NP, LBP, and migraine to improve prevention and treatment.

The objectives or our study were as follows: (i) to use the 2014 and 2020 European Health Interview Surveys for Spain (EHISS) to describe the prevalence of three types of pain, namely chronic neck pain (CNP), chronic low back pain (CLBP), and migraine or frequent headaches (MFH), in adults with asthma; (ii) to analyze how the prevalence of these three types of pain changes over time in adults with asthma; (iii) to ascertain whether sex and sociodemographic, clinical, and lifestyle variables were associated with the presence of these types of pain in asthma patients; and (iv) to analyze whether asthma is associated with the presence of any of the three types of pain studied by matching each individual with asthma to another without asthma and of identical age, sex, and region of residence.

## 2. Materials and Methods

### 2.1. Study Design and Data Source

A descriptive cross-sectional study and a retrospective case–control study were carried out. The data were obtained from the EHISS conducted in 2014 and 2020 (EHISS-2014 and EHISS-2020, respectively). In 2008, the European Commission promoted the European Health Survey based on representative population surveys of the European Union member states [21].

Face-to-face personal interviews were conducted at the participant’s home from January to December 2014 for EHISS-2014 and from July 2019 to July 2020 for EHISS-2020 [21,22]. During the last months of EHISS-2020 (March to July 2020), the information was collected over the phone owing to the onset of the COVID-19 pandemic [22]. The study variables used in this research come from questions with the same wording in both EHISS-2014 and EHISS-2020. More details on the EHISS are available online [23].

### 2.2. Study Population and Matching Method

The study included individuals over 17 years of age. We identified the interviewed individuals with and without asthma based on the question: “Has your doctor told you that you are suffering from asthma?” Those with a positive response were defined as “cases” for our study. For each “case”, an interviewee included in the same survey who lived in the same region, had an identical age and sex, and who would have answered “no” to the previous question was selected; this person was considered a “matched control”. If more than one control was available for a case, the selection was conducted randomly.

The total initial sample sizes of EHISS-2014 and EHISS-2020 were 22,843 (22,321 aged ≥18 years) and 22,072 (21,569 aged ≥18 years), respectively. Of these samples, 1312 in year 2014 and 1151 in year 2020 self-reported that their doctor had told them that they were suffering from asthma. We matched 1275 (97.7%) participants with asthma with 1275 non-asthmatic controls in EHISS-2014 and 1115 cases (96.9%) with 1115 controls in EHISS-2020.

### 2.3. Study Variables

The questions and answers used to identify the participants in EHISS-2014 and EHISS-2020 with CNP, CLBP, and MFH are shown in Table S1. Those participants with an affirmative answer to CNP and/or CLBP were considered “spinal pain” sufferers.

The EHISS define chronic pain as that which the patient reports having had for at least six consecutive months [23].

The study covariates of the study are detailed in Table S1.

### 2.4. Statistical Analysis

The prevalence of pain types in people with and without asthma was estimated for the two surveys and according to the covariates.

We provide for quantitative variable means with standard deviations and for qualitative variable total and relative frequencies expressed with percentages.

To compare the unmatched samples, the *t* test for quantitative and the chi-square test for qualitative variables were applied, respectively. For comparisons between the matched cases and the controls, paired *t* tests and McNemar’s test were used.

Multivariable logistic regression models were constructed, using CNP, CLBP, and MFH as dependent variables, to identify the study covariates associated with these pain localizations among the participants with asthma. We followed the recommendation of Hosmer et al. to construct the models, providing an adjusted odds ratio (OR) with its 95% confidence interval (95% CI) [24].

### 2.5. Sensitivity Analysis

The prevalence of the three types of pain was compared between the cases and controls after matching. To confirm the results, we constructed a logistic regression model, including the entire population in order to adjust for the effect of the presence of asthma on the remaining study covariates.

The statistical analysis was performed using STATA 14.0.

### 2.6. Ethical Considerations

We downloaded the microdata of EHISS-2014 and EHISS-2020 from the Ministry of Health of Spain (MHS) website [25]. Anyone can access and freely download the data, which are completely anonymized. The Spanish legislation does not require approval from an ethics committee because the data were collected by the Spanish National Statistics Institute (under the sponsorship of the MHS), which guarantees their anonymity, and they are freely and publicly accessible.

## 3. Results

Shown in Table 1 is the distribution of the study variables among the participants with asthma included in EHISS-2014 and EHISS-2020. In the people with asthma, no significant changes were observed between the years 2014 and 2020 for the crude prevalence of CNP (28.2% vs. 24.9%; *p* = 0.067), CLBP (31.3% vs. 30.5%; *p* = 0.686), MFH (14.3% vs. 12.1%; *p* = 0.100), spinal pain (38.6% vs. 36.3%; *p* = 0.234), and “any pain” (43.1% vs. 40.0%; *p* = 0.111). However, the intensity of pain was significantly lower in 2020 (*p* = 0.048).

### 3.1. Sex Differences in Prevalence of Pain among People with Asthma

**Figure 1:**
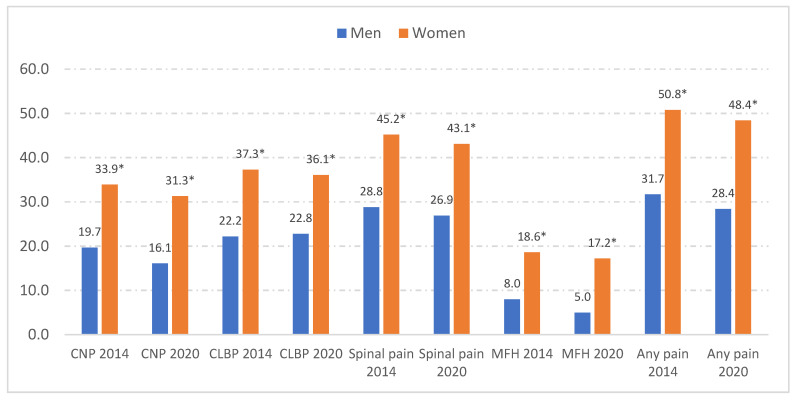
Sex differences in the prevalence of pain types among people with asthma interviewed in the 2014 and 2020 European Health Surveys for Spain (EHISS). CNP, chronic neck pain. CLBP, chronic low back pain. MFH, migraine or frequent headache. * *p* < 0.001 for the sex difference using the chi-square test.

As shown in Figure 1, the women with asthma reported a significantly higher prevalence of all types of pain than the men with asthma, in both surveys (all *p* < 0.001). Among the women, 50.8% in EHISS-2014 and 48.4% in EHISS-2020 reported at least one type of pain, with the prevalence being approximately 20% lower in the men. MFH was the type of pain with the lowest prevalence for both the women and men in both surveys, with rates of 18.6% and 17.2% for the women and 8.0% and 5.0% for the men in the 2014 and 2020 surveys, respectively. CLBP was reported by 37.3% of the women and 22.4% of the men in 2014 and 36.1% of the women and 22.8% of the men in 2020. The prevalence of CNP was higher among the women with asthma than among the men in 2014 (33.9% vs. 19.7%: *p* < 0.001) and in 2020 (31.3% vs. 16.1%; *p* < 0.001).

### 3.2. Prevalence of Pain among Cases and Matched Controls

Table 2 compares the prevalence of pain types between the patients with asthma and the controls without asthma, matched by EHISS year, sex, age, and region of residence according to sociodemographic variables and pain characteristics. After matching, the prevalence was significantly higher in the participants with asthma than in the controls for CNP (26.7% vs. 15.1%; *p* < 0.001), CLBP (30.9% vs. 17.5%; *p* < 0.001), and MFH (13.3% vs. 8.2%; *p* < 0.001).

The cases self-reported a higher prevalence of all types of pain than the controls when the populations were stratified by age, sex, and other sociodemographic variables (Table 2).

Table 3 displays the prevalence of pain types according to the clinical variables and the lifestyles of the patients with asthma and the matched controls. For all the variables analyzed in Table 3, CNP, CLBP, and MFH were reported in a significantly higher proportion of the individuals with asthma than in the controls.

### 3.3. Variables Associated with Self-Reported Presence of CNP, CLBP, and MFH in Asthma Patients

Among the individuals with asthma, CNP and CLBP increased significantly with age, with no differences found for MFH. For all three types of pain analyzed, more use of pain medication and lower educational level were significantly associated.

As seen in Table 3, among the patients with asthma the prevalence of CNP was notably high in those with concomitant mental diseases (49.5%), stroke (49.3%), cancer (47.4%), and heart diseases (46.3%). Among the individuals with asthma, CLBP was reported in 57.3% of those with stroke, 53.6% of those with mental diseases, and 52.2% of those with heart diseases. Finally, for MFH, the chronic diseases associated with the highest prevalence were mental illness (29.0%), stroke (25.3%), and cancer (23.4%), while self-rated “very good/good” health (7.6%) was associated with the lowest prevalence.

When analyzing lifestyle habits, we found that never engaging in physical activity during leisure time or having a BMI ≥ 30 was associated with a higher prevalence of the three types of pain analyzed.

As can be seen in Table 4, after adjusting for potential confounding factors using multivariable regression, the women with asthma had a significantly higher risk than the men with asthma of presenting CNP (OR 1.37; 95% CI 1.07–1.76), CLBP (OR 1.25, 95% CI 1.02–1.58), and MFH (OR 2.00; 95% CI 1.44–2.77). Self-rated “fair/poor/very poor” health was also associated with the three types of pain analyzed. Older age and concomitant MFH were predictors of CNP and CLBP. Mental illness and concomitant CLBP were associated with CNP and MFH. On the other hand, having COPD was associated with CNP, and younger age increased the likelihood of reporting MFH.

The use of pain medication and the presentation of concomitant CNP were associated with CLBP and MFH.

The logistic regression did not show significant changes in the prevalence of any of the pains investigated in the period from 2014 to 2020.

### 3.4. Sensitivity Analysis

Finally, as indicated in Supplementary Table S2, the results of the logistic regression analysis that included the entire study population confirmed the results obtained after matching. In this manner, we observed that having asthma increased the likelihood of CNP 1.45 times (OR 1.45; 95% CI 1.19–1.76), that of CLBP 1.37 times (OR 1.37; 95% CI 1.11–1.64), and that of MFH 1.19 times (OR 1.19; 95% CI 1.02–1.51).

## 4. Discussion

Our main findings are as follows: (1) No changes were observed between 2014 and 2020, with the prevalence of pain being higher in the asthma patients; (2) significant sex differences were found in the prevalence of these types of pain, with higher values and greater severity for the women with asthma than for the men with asthma; (3) experiencing any of the three types of pain was associated with poorer perception of health and a higher probability of reporting either of the other two; (4) in the people with asthma, multivariable analysis showed that female sex, “Fair/poor/very poor” self-reported health, and mental disorders were associated with all three types of pain analyzed.

Regarding the prevalence of pain, particularly back pain, most studies show a trend towards an increase in the near future, especially in relation to factors such as sedentary lifestyles, workload, and stress [26]. However, for the moment, the prevalence of pain has remained practically unchanged. This trend is reproduced in Spain, where the prevalence figures have not changed in the last 20 years. This was also true for migraine [27]. This is consistent with the results of our multivariate analysis, which confirmed no change in the prevalence of any of the three types of pains in the asthma patients between the 2014 and 2020 surveys. However, it is important to keep in mind that prevalence varies by geographic region and age group, and it is essential to be aware of these changes in order to implement effective pain prevention and management strategies in clinical practice.

In both surveys, we found a higher prevalence of all types of pain in the women than in the men with asthma. In addition, almost half of the women with asthma reported at least one type of pain; this was approximately 20% more than the men. As demonstrated in multiple studies in different countries, the prevalence of LBP is clearly higher in women (60% vs. 40%) [28]. Migraine, which affects 12.6% of the general population, is more than twice as prevalent in women (17.2% vs. 8%) [29], and NP affects more women in all age groups [12].

Sex differences in pain characteristics have also been described, with women being more sensitive to pain and at a higher risk of chronic pain than men. Endogenous opioid function and sex hormones influence sensitivity to pain among women [30,31,32].

The differences between men and women can be further explained by the greater likelihood that women will consult a doctor, use emergency services, and take prescription medication, as well as by their more frequent and earlier exposure to psychological distress. Furthermore, the unconscious biases of healthcare professionals themselves can affect the delivery of care, as women appear to be underdiagnosed or to receive less effective or aggressive pain treatment than men [30].

A Swedish study found that women experience more severe asthma than men. Moreover, women have a poorer quality of life than men at the same level of asthma severity [33].

We also observed that experiencing any of the three types of pain was associated with a higher probability of reporting either of the other two and that experiencing any of the three types of pain was associated with a poorer perception of health in terms of quality of life. For many years, it has been observed that patients who experience any type of pain self-report bad health more than those who do not experience pain [34].

Ergonomic and occupational factors, particularly heavy lifting, standing and sitting in a forward-leaning posture, and logged times sitting at a computer have been significantly associated with CLBP [28]. These types of behaviors lead to antalgic postures that trigger other types of pain, including NP.

Martin et al. [4] reported that people with asthma had a higher risk of developing migraine. It has also been shown that the occurrence of chronic migraine increases proportionally with the number of asthma symptoms [5]. Of note, MFH was the only one of the three types of pain analyzed in our study for which younger age was a predictive factor. As previously revealed, the prevalence of headache varies with age, increasing until around 40 years of age and decreasing thereafter in both sexes [9].

Regarding the role of comorbidities, we observed an association between mental illness, COPD, and pain. Asthma symptoms are not easy to cope with and may affect mental health [4].

In terms of marital status, widowed and divorced patients of both sexes are more likely to report low back pain. Loss of social support is associated with the risk of anxiety and depression, which may in turn be associated with musculoskeletal conditions such as LBP [28].

Another of the comorbidities associated with pain in our study was COPD, which shares specific causes with asthma and could be implicated as a predictor of certain types of pain, such as NP. De Miguel-Díez et al. recently found that among people with COPD, 40% reported CNP [35]. Similarly, in a COPD population, Bentsen et al. reported a high prevalence of NP [36]. As in asthma patients, hyperventilation has been shown to affect the respiratory muscles, causing alterations in the neck, shoulders, and thoracic region. Other studies have examined the relationship between diaphragm malfunction and COPD, reporting that the diaphragm decreases its ability to contribute to intra-abdominal pressure, which has an impact on spinal stiffness and, therefore, on spinal posture [37].

After matching and after the sensitivity analysis, we found the prevalence of the three types of pain to be higher in persons with asthma than in those without asthma. This finding may be very important, owing to the large number of people diagnosed with asthma in Spain.

Increasingly, epidemiological studies in patients with asthma are confirming a real increase in the prevalence of postural alterations, such as lumbar hyperlordosis, dorsal kyphosis, and anteversion and elevation of the shoulder [38].

In patients with persistent asthma, a strong association has also been recorded between deviations in posture and airway obstruction. The diaphragm has direct connections to the endothoracic fascia; so, lung inflation can lead to increased thoracic kyphosis [38,39].

Health surveys are useful for epidemiological investigation as the variables not frequently recorded in clinical records can be evaluated. The other strengths of our study are that a nationwide representative sample of people with asthma was available in two separate years and that we could control some of the confounding variables with the case–control design.

The reason why we only used two surveys, with an eight-year gap between them, was that the previous EHISS (the first EHISS) conducted in year 2009 had questions that were different to those of EHISS-2014 and EHISS-2020. Furthermore, in between EHISS-2014 and EHISS-2020, a Spanish National Health Survey was conducted in the year 2016; however, the questionnaire was also different; so, we could not join and compare the databases [22,40].

Our study is also subject to a series of limitations. First, a reverse causality bias must be considered due to the study design; therefore, associations, but not causality, can be established. Second, although our pain definitions have been used before, to our knowledge the questions included in the EHISS regarding pain have not yet been validated [34]. Third, only patients with a physician’s diagnosis of pain could be selected; so, the prevalence may be underestimated. Fourth, health surveys are affected by social desirability and memory bias.

## 5. Conclusions

Among the men and women with asthma, the prevalence of all the pain types was high and remained stable over time. The prevalence was higher and the severity was greater among the women with asthma than among the men with asthma. The prevalence of any pain was significantly higher in the people with asthma than in sex–age-matched individuals without asthma. Multivariable analysis showed that the variables associated with the reporting of the three types of pain in people with asthma were female sex, worse self-reported health, and self-reported mental illness.

## Figures and Tables

**Table 1:** Characteristics of the participants with self-reported asthma included in the years 2009 and 2020 European Health Surveys for Spain (EHISS).

Variable	Categories	EHISS 2014		EHISS 2020		
		n	%	n	%	*p*
Sex	Women	785	59.8	668	58.0	0.366
Age, Mean (SD)		52.6	(18.9)	54.1	(18.8)	0.165
Age groups (Years)	18–49	610	47.8	496	44.5	0.251
	50–69	370	29.0	340	30.5	
	70 or over	295	23.1	279	25.0	
Educational level	No studies/primary	745	56.8	629	54.6	0.210
	Secondary	211	16.1	216	18.8	
	High education	356	27.1	306	26.6	
Living with a partner	Yes	699	53.6	556	48.6	0.012
Chronic neck pain	Yes	370	28.2	287	24.9	0.067
Chronic low back pain	Yes	410	31.3	351	30.5	0.686
Spinal pain	Yes	507	38.6	418	36.3	0.234
Migraine or frequent headache	Yes	188	14.3	139	12.1	0.100
Any pain	Yes	566	43.1	460	40.0	0.111
Pain intensity	No pain	498	38.0	482	41.9	0.048
	Light	329	25.1	261	22.7	
	Moderate	265	20.2	249	21.6	
	Severe/extreme	220	16.8	159	13.8	
Use of pain medication	Yes	509	44.8	464	48.7	0.076
Self-rated health	Very good/good	624	47.6	555	48.2	0.744
	Fair/poor/very poor	688	52.4	596	51.8	
Diabetes	Yes	153	11.7	135	11.7	0.959
COPD	Yes	334	25.5	260	22.6	0.097
Heart diseases	Yes	170	13.0	165	14.3	0.320
Stroke	Yes	37	2.8	38	3.3	0.488
Cancer	Yes	67	5.1	70	6.1	0.292
High blood pressure	Yes	402	30.6	376	32.7	0.280
Mental disease	Yes	306	23.3	276	24.0	0.702
Physical activity in leisure time	No	557	42.6	507	44.1	0.443
Alcohol consumption	Yes	697	53.2	573	49.8	0.094
Active smoking	Yes	287	21.9	212	18.5	0.034
Body mass index	<25	545	41.6	456	39.8	0.392
	25–29.9	469	35.8	405	35.3	
	≥30	297	22.7	286	24.9	

*p* value, using chi-square test (proportions) or Student’s *t* test (means), for differences between the two EHISS. COPD, chronic obstructive pulmonary disease.

**Table 2:** Prevalence of self-reported pain types among cases with asthma and sex–age-matched controls without asthma according to sociodemographic and pain characteristics.

		CNP				CLBP					MFH		
		No Asthma n%		Asthma n%		No Asthma n%		Asthma n%			No Asthma n%	Asthma n%	
Sex ^d,e,f,g,h,i^	Men ^a,b,c^	85	8.4	182	18.0	125	12.4	227	22.5	30	3.0	66	6.5
	Women ^a,b,c^	288	19.8	475	32.7	307	21.1	534	36.8	171	11.8	261	18.0
Age groups ^d,e,f,g,h^	18–49 years ^a,b,c^	94	8.5	170	15.4	102	9.2	197	17.8	101	9.1	142	12.8
	50–69 years ^a,b,c^	140	19.7	244	34.4	164	23.1	287	40.4	65	9.2	110	15.5
	70 years or over ^a,b,c^	138	24.0	241	42.0	166	28.9	273	47.6	33	5.7	68	11.8
Educational level ^d,e,g,h,i^	No studies/primary ^a,b,c^	253	19.1	486	35.4	305	23.1	551	40.1	104	7.9	205	14.9
	Secondary ^a,b,c^	59	12.3	72	16.9	57	11.9	90	21.1	38	7.9	57	13.3
	High education ^a,b,c^	61	9.2	99	15.0	70	10.6	120	18.1	59	8.9	65	9.8
Living with a partner ^f^	No ^a,b,c^	192	15.9	305	25.6	213	17.6	350	29.3	81	6.7	167	14.0
	Yes ^a,b,c^	180	14.5	349	27.8	219	17.6	409	32.6	119	9.6	158	12.6
Pain intensity ^d,e,f,g,h,i^	No pain ^a,b,c^	56	4.2	82	8.4	73	5.5	92	9.4	45	3.4	42	4.3
	Light ^a,b,c^	92	16.6	141	23.9	109	19.7	174	29.5	44	8.0	67	11.4
	Moderate ^a,b,c^	117	32.5	219	42.6	127	35.3	240	46.7	60	16.7	104	20.2
	Severe/extreme ^a,c^	108	51.7	215	56.7	123	58.9	255	67.3	52	24.9	114	30.1
Use of pain medication ^d,e,f,g,h,i^	No ^a,b,c^	92	9.7	196	17.6	112	11.8	205	18.4	53	5.6	88	7.9
	Yes ^a,b,c^	233	34.7	434	44.6	279	41.6	510	52.4	121	18.0	223	22.9
Concomitant CNP ^e,f,h,i^	No ^b,c^	NA	NA	NA	NA	157	12.1	239	16.4	111	8.6	133	9.1
	Yes ^b,c^	NA	NA	NA	NA	234	72.0	476	75.6	63	19.4	178	28.3
Concomitant CLBP ^d,f,g,i^	No ^a,c^	91	7.4	154	11.2	NA	NA	NA	NA	100	8.1	128	9.3
	Yes ^c^	234	59.8	476	66.6	NA	NA	NA	NA	74	18.9	183	25.6
Concomitant MFH ^d,e,g,h^	No ^a,b^	262	18.1	452	25.5	317	22	532	30.0	NA	NA	NA	NA
	Yes ^a,b^	63	36.2	178	57.2	74	42.5	183	58.8	NA	NA	NA	NA

NA: Not applicable. CNP, chronic neck pain. CLBP, chronic low back pain. MFH, migraine or frequent headache. ^a^ *p* < 0.05 cases vs. controls with CNP. ^b^ *p* < 0.05 cases vs. controls with CLBP. ^c^ *p* < 0.05 cases vs. controls with MFH. ^d^ *p* < 0.05 for CNP among controls. ^e^ *p* < 0.05 for CLBP among controls. ^f^ *p* < 0.05 for MFH among controls. ^g^ *p* < 0.05 for CNP among cases. ^h^ Significant for CLBP among cases. ^i^ *p* < 0.05 for MFH among cases. McNemar’s test was used to compare proportions.

**Table 3:** Prevalence of self-reported pain types among cases with asthma and sex–age-matched controls without asthma according to sociodemographic variables and according to clinical and lifestyle variables.

		CNP				CLBP				MFH			
		No Asthma, n%		Asthma, n%		No Asthma, n%		Asthma, n%		No Asthma, n%		Asthma, n%	
Self-rated health ^d,e,f,g,h,i^	Fair/poor/very poor ^a,b,c^	241	33.3	510	43.3	288	39.8	582	49.4	104	14.4	230	19.5
	Very good/good ^a,b,c^	132	7.6	147	11.4	144	8.3	179	13.9	97	5.6	97	7.6
Diabetes ^d,e,g,h^	No ^a,b,c^	321	14.2	543	25.0	366	16.2	616	28.3	185	8.2	284	13.1
	Yes ^a,b,c^	52	25.1	114	39.6	66	31.9	145	50.3	16	7.7	43	14.9
COPD ^d,e,f,g,h,i^	No ^a,b,c^	339	14.2	403	21.6	394	16.6	474	25.4	185	7.8	218	11.7
	Yes ^a,b,c^	34	40.5	254	42.8	38	45.2	287	48.3	16	19.0	109	18.4
Heart diseases ^d,e,g,h,i^	No ^a,b,c^	293	13.2	502	23.6	340	15.3	586	27.5	179	8.1	269	12.6
	Yes ^a,b,c^	80	32.4	155	46.3	92	37.2	175	52.2	22	8.9	58	17.3
Stroke ^d,e,g,h,i^	No ^a,b,c^	356	14.8	620	26.0	413	17.1	718	30.1	197	8.2	308	12.9
	Yes ^a,c^	17	33.3	37	49.3	19	37.3	43	57.3	4	7.8	19	25.3
Cancer ^e,g,h,i^	No ^a,b,c^	352	14.9	592	25.5	406	17.1	692	29.8	192	8.1	295	12.7
	Yes ^a,b,c^	21	22.1	65	47.4	26	27.4	69	50.4	9	9.5	32	23.4
High blood pressure ^d,e,g,h,i^	No ^a,b,c^	228	12.3	342	20.3	249	13.4	394	23.4	143	7.7	190	11.3
	Yes ^a,b,c^	145	23.9	315	40.5	183	30.1	367	47.2	58	9.6	137	17.6
Mental disease ^d,e,f,g,h,i^	No ^a,b,c^	253	11.9	369	19.6	289	13.6	449	23.9	132	6.2	158	8.4
	Yes ^a,b,c^	120	35.7	288	49.5	143	42.6	312	53.6	69	20.5	169	29.0
Physical activity in leisure time ^d,e,f,g,h,i^	Never ^a,b,c^	195	20.9	383	36.0	222	23.8	433	40.7	104	11.2	171	16.1
	Occasionally or frequent ^a,b,c^	178	11.6	272	19.5	210	13.7	327	23.4	97	6.3	156	11.2
Alcohol consumption ^d,e,f,g,h,i^	No ^a,b,c^	219	18.4	394	33.1	253	21.2	448	37.6	111	9.3	210	17.6
	Yes ^a,b,c^	153	12.1	262	20.6	177	14	312	24.6	90	7.1	117	9.2
Active smoking ^d,e,g,h^	No ^a,b,c^	301	15.8	546	27.8	353	18.5	647	33.0	150	7.9	259	13.2
	Yes ^a,b,c^	72	13.1	110	22	79	14.3	113	22.6	51	9.3	68	13.6
Body mass index ^d,e,f,g,h,i^	<25 ^a,b,c^	145	12	212	21.2	163	13.5	241	24.1	102	8.5	125	12.5
	25–29.9 ^a,b,c^	137	16.1	236	27	166	19.5	280	32.0	54	6.3	114	13.0
	≥30 ^a,b,c^	90	22.4	208	35.7	103	25.6	238	40.8	44	10.9	87	14.9

CNP, chronic neck pain. CLBP, chronic low back pain. MFH, migraine or frequent headache. ^a^ *p* < 0.05 cases vs. controls with CNP. ^b^ *p* < 0.05 cases vs. controls with CLBP. ^c^ *p* < 0.05 cases vs. controls with MFH. ^d^ *p* < 0.05 for CNP among controls. ^e^ *p* < 0.05 for CLBP among controls. ^f^ *p* < 0.05 for MFH among controls. ^g^ *p* < 0.05 for CNP among cases. ^h^ Significant for CLBP among cases. ^i^ *p* < 0.05 for MFH among cases. McNemar’s test was used to compare proportions. COPD, chronic obstructive pulmonary disease.

**Table 4:** Variables associated with the three types of pain analyzed among people with asthma identified with multivariable logistic regression.

		CNP	CLBP	MFH
		OR (95% CI)	OR (95% CI)	OR (95% CI)
Sex	Men	1	1	1
	Women	1.37 (1.07–1.76)	1.25 (1.02–1.58)	2.00 (1.44–2.77)
Age groups	18–49 years	1	1	1
	50–69 years	1.41 (1.05–1.88)	1.76 (1.31–2.35)	0.41 (0.29–0.58)
	70 years or over	1.64 (1.20–2.23)	1.52 (1.11–2.09)	0.21 (0.14–0.32)
Self-rated health	Very good/good	1	1	1
	Fair/poor/very poor	0.50 (0.38–0.65)	0.47 (0.36–0.61)	0.74 (0.0.51–0.97)
Diabetes	No	NSDM	1	NSDM
	Yes	NSDM	1.39 (1.00–1.93)	NSDM
COPD	No	1	NSDM	NSDM
	Yes	1.31 (1.01–1.70)	NSDM	NSDM
Mental disorder	No	1	NSDM	1
	Yes	1.55 (1.19–2.02)	NSDM	2.73 (2.05–3.63)
Use of pain medication	No	NSDM	1	1
	Yes	NSDM	2.46 (1.91–3.16)	2.19 (1.61–2.98)
Concomitant CNP	No	NA	1	1
	Yes	NA	9.81 (7.67–12.54)	2.44 (1.76–3.37)
Concomitant CLBP	No	1	NA	1
	Yes	10.06 (7.95–12.73)	NA	1.65 (1.19–2.30)
MFH	No	1	1	NA
	Yes	2.39 (1.74–3.28)	1.67 (1.20–2.32)	NA
YEAR	2020	0.81 (0.65–1.02)	1.05 (0.83–1.33)	0.81 (0.62–1.06)

CNP, chronic neck pain. CLBP, chronic low back pain. MFH, migraine or frequent headache. COPD, chronic obstructive pulmonary disease. NA, not adequate. NSDM, not significant in the definitive model. OR, odds ratio. CI, confidence interval.

## Data Availability

According to the contract signed with the Spanish Ministry of Health and Social Services, which provided access to the database from European Health Survey for Spain, the authors cannot share the database with any other investigator, and they have to destroy the database once the investigation has concluded. Consequently, the authors cannot upload the database to any public repository. However, any investigator can apply for access to the database by filling out the questionnaire available at http://www.msssi.gob.es/estadEstudios/estadisticas/estadisticas/estMinisterio/SolicitudSNHSdocs/Formulario_Peticion_Datos_SNHS.pdf (accessed on 24 February 2023). All other relevant data are included in the paper.

## Supplementary Materials

The following supporting information can be downloaded at: https://www.mdpi.com/article/10.3390/jcm12227107/s1, Table S1: Questions and possible answers of the 2014 and 2022 EHISS questionnaires used to create the study variables, Table S2: Sensitivity analysis.
