# Supplementary material for: Asthma Is Associated with Back Pain and Migraine—Results of Population-Based Case–Control Study
Source: J Clin Med. 2023 Nov 15;12(22):7107. doi: 10.3390/jcm12227107 (PMC10671864; doi:10.3390/jcm12227107)
Supplement: Supplementary file 1 [file jcm-12-07107-s001.zip › jcm-2723874-supplementary.pdf]

Table S1. Questions and possible answers of the 2014 and 2022 EHISS questionnaires used to create the study variables.

**Questions**	**Description and answer**	**Variables**	**Categories**
Which is your sex?	1. Men<br>2. Women	Sex	1. Men<br>2. Women
How old are you?	Age in years	Age groups	1. 18-49<br>2. 50-69<br>3. 70 or over
Has your doctor told you that you are suffering from asthma?	1.Yes<br>2.No	Asthma	1. Case<br>2. Control
Has your doctor told you that you are suffering from chronic neck pain (6 or more months)?	1.Yes<br>2.No	*Chronic neck pain*	1. Yes<br>2. No
Has your doctor told you that you are suffering from chronic low back pain (6 or more months)?	1.Yes<br>2.No	*Chronic low back pain*	1. Yes<br>2. No
Has your doctor told you that you are suffering from migraine or frequent headaches?	1.Yes<br>2.No	*Migraine or frequent headaches*	1. Yes<br>2. No
What level of education have you completed?	1. Does not know how to read or write<br>2. Incomplete primary education<br>3. Complete primary education<br>4. First stage of Secondary Education, with or without a qualification<br>5. Elementary Spanish Upper Secondary Education<br>6. Upper secondary education<br>7. Intermediate vocational training or equivalent<br>8. Advanced vocational training or equivalent<br>9. University studies or equivalent<br>10. Over university (master, PhD….)	Educational level	1. No studies/Primary: Options 1 to 3<br>2. Secondary: Options 4 to 8<br>3. High education: Options 9 and 10
What is your marital status?	1. Single<br>2. Married<br>3. Widower<br>4. Separated<br>5. Divorced	Living with a partner	1. Yes: Option 2<br>2. Nor: options 1, 3, 4 and 5
"Over the last four week, what intensity of pain have you suffered?	1 None,<br>2. Very light,<br>3. Light,<br>4. Moderate,<br>5. Severe<br>6. Extreme.	Pain intensity	1, No pain. Option 1<br>2. Light. Options 2 and 3<br>3. Moderate. Option 4<br>4. Severe/extreme. Options 5 and 6
1. Next I am going to read you a list of types of medications, please tell me which one or more of them have you taken in the last 2 weeks?	A list of 23 medications is read the person interviewed including<br>1 pain medication	Use of pain medication	1. Yes<br>2. No

Table S1. Questions and possible answers of the 2014 and 2022 EHISS questionnaires used to create the study variables. (Continued)

**Questions**	**Description and answer**	**Variables**	**Categories**
In the past twelve month, how is your perception of your general health status?	1. Very good<br>2. Good<br>3. Fair<br>4. Bad<br>5. Very bad	Self-rated health	1. Very good/good: Options 1 and 2<br>2. Fair/poor/very poor: Options 3 to 5
Has your doctor told you that you are suffering from COPD*?*	1.Yes<br>2.No	COPD	1. Yes<br>2. No
Has your doctor told you that you are suffering from diabetes*?*	1.Yes<br>2.No	Diabetes	1. Yes<br>2. No
Has your doctor told you that you are suffering from stroke?	1.Yes<br>2.No	*Stroke*	1. Yes<br>2. No
Has your doctor told you that you are suffering from cancers?	1.Yes<br>2.No	Cancer	1. Yes<br>2. No
Has your doctor told you that you are suffering from anxiety or depression?	1.Yes<br>2.No	Mental disease	1. Yes<br>2. No
Has your doctor told you that you are suffering from High blood pressure?	1.Yes<br>2.No	*High blood pressure*	1. Yes<br>2. No
Which of these possibilities best describes how often you do some physical activity in your free time?	1. I don't exercise. I occupy my free time almost completely sedentary<br>2. I do some occasional physical or sports activity<br>3. I do physical activity several times a month<br>4. I do sports or physical training several times a week	Sedentary lifestyle	1. Yes: Option 1<br>2. No: Option 2 to 4
During the past 12 months, how often have you had alcoholic beverages of any kind (i.e. beer, wine, spirits, distilled and mixed drinks, or other alcoholic beverages)?	1. Daily or almost daily<br>2. 5-6 days per week<br>3. 3-4 days per week<br>4. 1-2 days per week<br>5. 2-3 days in a month<br>6. Once a month<br>7. Less than once a month<br>8. Not in the last 12 months, have I stopped drinking<br>9. Never or just a few sips to taste it throughout life	Alcohol consumption	1. Yes: Options 1 to 6<br>2. No: Option 7 to 9
Could you tell me if you smoke?	1. Yes, I smoke daily<br>2. Yes, I smoke, but not daily<br>3. I don't currently smoke but have smoked before<br>4. I neither smoke nor have I ever smoked regularly	Active smoking	1. Yes: Options 1 and 2<br>2. No: Options 3 and 4
1. Could you tell me how tall you are, approximately, without shoes?<br>2. Could you tell me your weight, approximately, without shoes and clothes?	Body mass index is calculated with the formulae: Weight in kg/ (Height in meters)^2	Body mass index	1. <25<br>2, 25-29.9<br>3. ≥30

COPD, Chronic obstructive pulmonary disease.

Table S2. Sensitivity analysis.

		CNP	CLBP	MFH
		OR (95% CI)	OR (95%CI)	OR (95%CI)
Sex	Man	1	1	1
	Women	1.45 (1.18-1.79)	1.18 (1.02-1.40)	2.24 (1.72-2.92)
Age groups	18-49 years	1	1	1
	50-69 years	1.39 (1.09-1.77)	1.67 (1.32-2.11)	0.45 (0.34-0.58)
	70 years or over	1.40 (1.09-1.80)	1.41 (1.10-1.82)	0.22 (0.16-0.29)
Self-rated health	Very good/good	1	1	1
	Fair/poor/very poor	0.57 (0.46-0.71)	0.48 (0.39-0.60)	0.76 (0.59-0.99)
COPD	No	1	1	1
	Yes	1.32 (1.04-1.69)	1.28 (1.01-1.63)	1.32 (1.00-1.74)
Mental disorder	No	1	1	1
	Yes	1.36 (1.09-1.68)	1.30 (1.05-1.61)	2.25 (1.79-2.84)
Use of pain medication	No	1	1	1
	Yes	1.48 (1.20-1.82)	2.51 (2.06-3.05)	2.39 (1.88-3.05)
Concomitant CNP	No	NA	1	1
	Yes	NA	10.07 (8.30-12.23)	1.90 (1.47-2.47)
Concomitant CLBP	No	1	NA	1
	Yes	10.11 (8.33-12.28)	NA	1.55 (1.19-2.03)
Concomitant MFH	No	1	1	NA
	Yes	1.88 (1.45-2.45)	1.54 (1.18-2.00)	NA
Year	2020	0.87 (0.68-1.03)	0.96 (0.80-1.15)	0.83 (0.67-1.31)
Asthma	No	1	1	1
	Yes	1.45 (1.19-1.76)	1.37 (1.11-1.64)	1.19 (1.02-1.51)

COPD, Chronic obstructive pulmonary disease. NA, Not adequate. NIFM, Not included in final the model. OR, Odds ratios estimated using multivariable unconditional logistic regression. CI, Confidence interval.
